# Transforming growth factor beta–related proteins promote axonal regeneration of injured dorsal root ganglion neurons

**DOI:** 10.4103/NRR.NRR-D-24-01427

**Published:** 2025-05-06

**Authors:** Yinying Shen, Peng Yang, Wenyu Dai, Xiaosong Gu, Sheng Yi

**Affiliations:** Medical School of Nantong University, Key Laboratory of Neuroregeneration of Jiangsu and Ministry of Education, Co-innovation Center of Neuroregeneration, NMPA Key Laboratory for Research and Evaluation of Tissue Engineering Technology Products, Nantong University, Nantong, Jiangsu Province, China

**Keywords:** activin A, angiopoietin 2, axon elongation, axonal regeneration, dorsal root ganglion, E2f2, Gper1, growth factor, neurite outgrowth, neuron

## Abstract

Dorsal root ganglia neurons gradually lose their axonal regeneration ability during development and aging. To explore molecules that enhance axonal regeneration, we screened growth factors with differential gene expression patterns in the dorsal root ganglias of young adult and aged animals following sciatic nerve injury. In young adult animals, two transforming growth factor beta-related factors, activin A and angiopoietin 2, were found to be upregulated post nerve injury. Treatment of isolated dorsal root ganglia explants and cultured dorsal root ganglia neurons of neonatal and young adult rats with recombinant activin A or angiopoietin 2 protein stimulated neurite outgrowth and axonal elongation. The administration of recombinant activin A or angiopoietin 2 protein to sciatic nerve crush-injured dorsal root ganglias also supported the growth of sensory neurons and facilitated nerve regeneration in both young adult and aged rats. Using RNA sequencing, we characterized genetic changes in dorsal root ganglia neurons following recombinant activin A or angiopoietin 2 treatment, revealing the unique mechanisms of these transforming growth factor beta–related factors. Recombinant activin A elicited changes in the gene expression of cytoskeleton-related Gper1 and activated extracellular signal-regulated kinase signaling, while angiopoietin 2 increased the expression of the transcription factor gene *E2f2*. Our identification of activin A and angiopoietin 2 as crucial promotional factors of axonal regeneration may guide future therapeutic strategies for the treatment of nerve injury.

## Graphical abstract

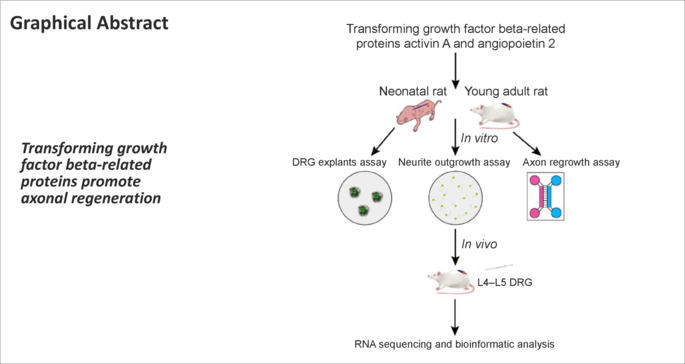

## Introduction

The intrinsic growth ability of injured neurons is an important basis for effective axon elongation and nerve regeneration (He and Jin, 2016). In higher mammals, axonal growth competency progressively diminishes in the postnatal stage and declines further with aging, leading to impaired regeneration outcomes (Mahar and Cavalli, 2018; Hilton et al., 2022). Exploring the genetic foundations underlying the nervous system of mammals at different ages may help to identify factors essential for axonal growth and regeneration. For example, a comparison of injured dorsal root ganglia (DRG) in young adult and aged mice revealed that aging-dependent enrichment of CXCR5^+^CD8^+^ T cells activates neuronal caspase 3 and hinders nerve regeneration (Zhou et al., 2022). Notably, besides the differences in immune cells, many growth factor genes exhibit dissimilar expression patterns among mice of different ages following sciatic nerve injury (SNI) (Zhou et al., 2022).

Growth factors are morphogenetic proteins that regulate numerous critical cellular functions and coordinate the steps of multi-stage tissue repair and wound healing (Shan and Wu, 2024; Tuffaha and Lee, 2024). Many growth factors show upregulated expression in DRGs following peripheral nerve injury (Terada et al., 2018; Shen et al., 2020). For instance, the expression of brain-derived neurotrophic factor (BDNF) is upregulated in the injured DRGs of both young adult and aged mice (Zhou et al., 2022). Elevated BDNF contributes to neurogenesis, neuron survival, and axonal regeneration (Heng et al., 2024; Zeng et al., 2024). However, the expression patterns of certain other growth factor genes, including those that encode proteins related to transforming growth factor beta (TGF-β) (e.g., activin A [*Inhba*] and angiopoietin 2 [*Angpt2*]), are altered in young adult animals while remaining unaffected in aged animals (Zhou et al., 2022).

The TGF-β signaling pathway is a key player in axonal regeneration (Vidal et al., 2013; Chandran et al., 2016). Activin A (ActA) belongs to the TGF-β superfamily and functions as a ligand in the TGF-β signaling pathway, binding to the ActA receptor and activating the phosphorylation of Smad2/3 (Xiao et al., 2022). Angiopoietin 2 (Ang2) stimulates TGF-β expression, while TGF-β, in turn, induces Ang2 expression (Schulz et al., 2011; Chang et al., 2014). The distinct expression patterns of *Inhba* and *Angpt2* in the injured DRGs of young adult versus aged animals indicate that ActA and Ang2 may facilitate axonal regeneration, with unaltered expression of these genes possibly contributing to regeneration failure in aged animals. The purpose of this study was to investigate the effects of ActA and Ang2 on axonal regeneration of DRG neurons and explore their potential molecular mechanisms.

## Methods

### Animals

The animal research was approved by the Ethics Committees of Experimental Animals, Jiangsu Province, China (approval ID: S20231219-061; December 19, 2023). All experiments were designed and reported in accordance with the Animal Research: Reporting of *In Vivo* Experiments (ARRIVE) guidelines (Percie du Sert et al., 2020). A total of 96 specific-pathogen free (SPF) Sprague-Dawley rats with the following sets of characteristics were purchased from the Animal Center of Nantong University (Animal License Nos. SCXK [Su] 2014-0001 and SYXK [Su] 2012-0031): 1) **~**1-day-old, body weight 15–20 g; 2) young adult (8 weeks), male, bodyweight 200–220 g; and 3) middle-aged (10 months), male, bodyweight 700–800 g. The rats were bred under SPF conditions at temperatures ranging from 20°C to 24°C and relative humidity from 45% to 65%, and were housed under a 12/12-hour light/dark cycle and provided with free access to water and a standard chow diet. Euthanization was carried out via post-freezing decapitation of 1-day-old neonatal rats and CO_2_ asphyxiation of adult rats. Every effort was made to minimize any potential suffering.

### Preparation and treatment of dorsal root ganglia explants

DRGs isolated from a total of 12 neonatal rats were dissociated by placement on glass coverslips of Petri dishes containing Dulbecco’s modified Eagle’s medium supplemented with 1% penicillin and streptomycin. Following trimming, DRG explants were covered with neuron culture medium containing 2% B27 Supplement (Stemcell Technologies, Vancouver, Canada), 1% L-glutamine (Beyotime), and 1% penicillin and streptomycin (Beyotime). Bovine serum albumin (BSA) solvent, or 5 ng/mL concentrations of recombinant human/mouse/rat activin A (rActA, Cat# ab151687; Abcam, Cambridge, MA, USA) or recombinant human angiopoietin 2 (rAng2, Cat# ab123226, Abcam) dissolved in 0.1% BSA, were added to the neuron culture medium. Thirty-six hours later, the DRG explants were fixed with 4% paraformaldehyde, blocked with immunol staining blocking buffer (Beyotime, Shanghai, China), and incubated with rabbit anti-Tuj1 antibody (1:1000 dilution, Cat# ab18207, RRID: AB_444319; Abcam) for 18 hours at 4°C. Next the mixture was incubated with the secondary antibody, donkey anti-rabbit Alexa Fluor^TM^ 488 (1:400, Cat# A-21206, RRID: AB_2535792; Invitrogen, Carlsbad, CA, USA) for 2 hours at room temperature. Tuj1 can be used to detect axon growth of DRG explants (Zhao et al., 2021), while the number of axonal intersections and area under the curve (AUC) serve as quantitative indicators of axonal growth (Livni et al., 2019). Whole DRG tissue explants and surrounding axons were imaged using a Zeiss microscope. Fiji software 1.52 (National Institutes of Health, Bethesda, MD, USA) (Schindelin et al., 2012) was used to measure the number of intersections and GraphPad Prism 6.02 (GraphPad Software, Boston, MA, USA, www.graphpad.com) was used to measure the AUC.

### Isolation, culture, and treatment of neonatal and young adult rat dorsal root ganglia neurons

Primary DRG neurons were dissected out from nine neonatal rats and 10 young adult rats, cut into small pieces, and then subjected to enzyme digestion. Neonatal rat DRGs were digested with 3 mg/mL collagenase (Sigma, St. Louis, MO, USA) for 30 minutes at 37°C, followed by 0.25% trypsin for 10 minutes at 37°C, centrifuged to remove the supernatant, re-suspended in 15% BSA, and then centrifuged to collect neonatal DRG neurons. Young adult rat DRGs were digested with 3 mg/mL collagenase for 90 minutes at 37°C, followed by 0.25% trypsin for 5 minutes at 37°C, centrifuged to remove the supernatant, re-suspended in 15% BSA twice, and then centrifuged to collect adult DRG neurons.

Isolated neonatal and young adult rat DRG neurons were plated onto cell culture dishes precoated with 100 mg/mL poly-L-lysine and cultured in neurobasal medium in a humidified 5% CO_2_ incubator at 37°C. For recombinant protein treatment, 5 ng/mL rActA or rAng2 dissolved in 0.1% BSA was added to the neuron culture medium.

### *In vitro* neurite outgrowth assay

Primary neonatal and adult rat DRG neurons cultured on poly-L-lysine-coated cell culture dishes were directly exposed to recombinant protein for 24 hours, fixed with 4% paraformaldehyde, and then immunostained with anti-Tuj1 antibody to visualize neuronal soma and neurites. ImageJ software 1.51 (National Institutes of Health) (Schneider et al., 2012) was used to measure the length of each neurite.

### *In vitro* axon regrowth assay

Two-compartment microfluidic chambers (Cat# SND150, Xona 2-compartment SND 150; Xona Microfluidics LLC, Los Angeles, CA, USA) were equally coated with poly-L-lysine. The somal compartment was filled with recombinant protein-containing neuron culture medium; the axonal compartment was filled with the same medium without recombinant protein. Adult rat DRG neurons were resuspended in neuron culture medium, seeded onto the somal compartment, and cultured for 48 hours to allow axons to grow into the axonal compartment along the microgroove barrier. Neuronal axons in the axonal compartment were then cut and removed using 0.08-MPa vacuum suction three times for 20 seconds each time. Dissected neuronal axons were continuously cultured for 36 hours and fixed at the same time prior to immunocytochemistry. ImageJ software was used to measure the length of regrown axons.

### *In vivo* treatment with recombinant protein

A total of 14 young and 12 middle-aged rats were subjected to sciatic nerve crush injury as previously described, with some modifications (Yi et al., 2015). Briefly, after anesthetization, the left sciatic nerves of adult rats were exposed by a small skin incision; sciatic nerves at 10 mm above the bifurcation into the tibial and common fibular nerves were then crushed using forceps within 30 seconds. Rats that underwent sciatic nerve crush injury were randomly and equally divided into four groups and given 100 ng/mL rActA or rAng2 (dissolved in 0.1% BSA), or BSA control (0.1% BSA). Recombinant protein or BSA control was administered via gradual injection into the L4 and L5 DRGs using a microsyringe (2 μL/DRG) after making a small skin incision at the midline of the lower back to expose these DRGs. Injured rat sciatic nerves were removed at 3 days post crush injury, fixed with 4% paraformaldehyde, cryoprotected in 30% sucrose, and cut into 10-μm thick nerve sections using a cryostat (Leica Microsystems, Bensheim, Germany). Nerve sections were immunostained with the primary antibody rabbit anti-stathmin-2/STMN2 (SCG10; 1:500, Cat# NBP1-49461, RRID: AB_10011569; Novus Biologicals, Denver, CO, USA) for 18 hours at 4°C, followed by the secondary antibody, donkey anti-rabbit Alexa Fluor^TM^ 488 (1:400) for 2 hours at room temperature. SCG10 was used as a marker to specifically label the regenerated sensory axons in the sciatic nerve (Yang et al., 2020). ImageJ software was used to measure the length of regenerated nerve fibers.

### RNA sequencing and bioinformatic analysis

For the discovery of upstream growth factors, significantly differentially expressed genes (DEGs) with a false discovery rate < 0.05 that were identified in the injured DRGs of young adult and aged mice in a previous study (Zhou et al., 2022) were uploaded to the Ingenuity Pathway Analysis software program (http://ingenuity.com/index.htm/; Ingenuity Systems Inc., Redwood City, CA, USA). The Ingenuity Pathway Analysis upstream regulator analysis tool was then applied to screen for DEGs encoding upstream growth factors.

To identify rActA- or rAng2-induced genetic changes, total RNA was extracted from adult rat DRG neurons treated with rActA or rAng2 protein or BSA control using a TRIzol reagent kit (Invitrogen) following the manufacturer’s instructions. RNA quality was determined using an Agilent 2100 Bioanalyzer (Agilent Technologies, Palo Alto, CA, USA). RNA sequencing was then performed on a HiSeqTM 4000 by Genedenovo Biotechnology Co., Ltd. (Guangzhou, China). Differential expression testing was performed using edgeR (Robinson et al., 2010). Genes with a fold change ≥ 2 or ≤ –2 and a *P* value < 0.05 were screened as DEGs. DEGs were mapped to the Gene Ontology (GO) database (http://www.geneontology.org/; Ashburner et al., 2000) and the Kyoto Encyclopedia of Genes and Genomes (KEGG) database (https://www.kegg.jp/; Kanehisa and Goto, 2000) to identify significantly enriched GO terms and KEGG pathways, respectively. Target genes of the transcription factor E2F2 were predicted using the Gene Transcription Regulation Database (GTRD; http://gtrd.biouml.org/; Kolmykov et al., 2021) and JASPAR database (https://jaspar.genereg.net/; Castro-Mondragon et al., 2022). The binding of E2F2 to binding sites in the target genes encoding POP7 homolog ribonuclease P/MRP subunit (*Pop7*), cyclin B1 (*Ccnb1*), and glutaryl-CoA dehydrogenase (*Gcdh*) was identified using the motif-based sequence analysis tool FIMO from the MEME Suite (http://meme-suite.org/tools/fimo) (Bailey et al., 2009).

### Reverse transcription-polymerase chain reaction analysis

Total RNA extracted from rActA-, rAng2-, or control-treated young adult rat DRG neurons was treated with amplification-grade DNase I (Yishan Biotechnology Co., Shanghai, China), quantified with a Nanodrop 1000 spectrophotometer (NanoDrop Technologies, Wilmington, DE, USA), and then converted into cDNA using the HiScript III RT SuperMix for qPCR (Vazyme, Nanjing, China). The cDNA was amplified in a 10-μL reaction containing SYBR Mix (Vazyme) and RT-PCR was conducted using an ABI StepOne system (Applied Biosystems, Foster City, CA, USA). The relative expression levels of target genes encoding G protein-coupled estrogen receptor 1 (*Gper1*) and *E2f2* were calculated using the 2^–ΔΔCT^ method (Francio et al., 2023), employing glyceraldehyde 3-phosphate dehydrogenase (*GAPDH*) as the reference gene. The sequences of the forward and reverse primers were as follows: (*Gper1*) 5′-CAG CAC AAG ATG TTG GCG TAG-3′ (forward) and 5′-AGA GGT CCC CAG TGA GGT TC-3′ (reverse); *E2f2* 5′-CTT CGC TTT ACA CGC AGA CG-3′ (forward) and 5′-CCC AGG AGA CTT AGG GGT TTT-3′ (reverse); *GAPDH* 5′-ACA GCA ACA GGG TGG TGG AC-3′ (forward) and 5′-TTT GAG GTG CAG CGA ACT T-3′ (reverse). All primers were synthesized by Sangon Biotech (Shanghai, China).

### Western blot assay

Total protein was extracted from recombinant protein- or control-treated adult rat DRG neurons using radio immunoprecipitation assay lysis buffer (Beyotime) containing protease and phosphatase inhibitors. Protein concentration was quantified using an enhanced BCA protein assay kit (Beyotime). Equal amounts of protein samples were resolved by 10% sodium dodecyl sulfate-polyacrylamide gel electrophoresis (Beyotime) and transferred to polyvinylidene fluoride membranes (Millipore, Burlington, MA, USA). The membranes were blocked with 5% BSA, incubated with rabbit anti-phospho-p44/42 mitogen-activated protein kinase (MAPK; phosphorylated extracellular signal-regulated kinase [Erk]1/2) and p44/42 MAPK (ERK1/2) antibodies (1:1000, Cell Signaling Technology, Boston, MA, USA, Cat# 4695T and 4370T, RRID: AB_10829379 and AB_2067555) at 4°C overnight. The blots were then incubated with horseradish peroxidase-conjugated secondary antibodies (1:1000, Cat# A0208, RRID: AB_2892644; Beyotime) for 2 hours at room temperature and developed with an ECL Western Blotting Detection Kit (Tanon, Shanghai, China). ImageJ software was used to quantify the densities of protein bands.

### Statistical analysis

All quantitative data are expressed as means ± the standard error of the mean. Using GraphPad Prism software, an unpaired two-tailed Student’s *t*-test or two-way analysis of variance, followed by Sidak’s multiple comparisons test, was employed to assess significance. A *P* value < 0.05 was considered statistically significant.

## Results

### Transforming growth factor beta–related genes *Inhba* and *Angpt2* are elevated in the dorsal root ganglias of adult rats post-sciatic nerve injury

**Figure 1:**
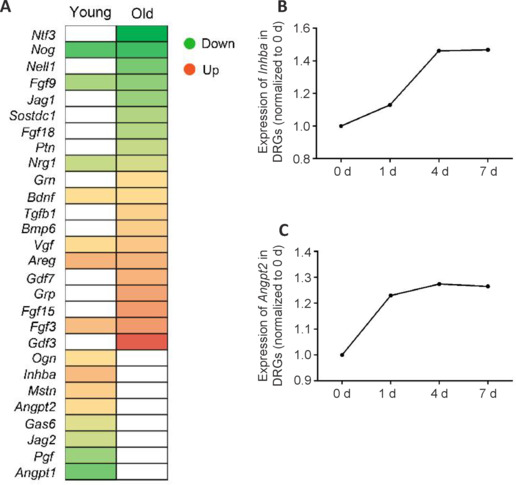
Differential expression of TGF-β-related genes *Inhba* and *Angpt2* Identified in rodent DRGs following sciatic nerve injury. (A) Relative expression levels of differentially expressed genes coding for upstream growth factors in the injured DRGs of young adult and aged mice in a previous study. (B, C) Temporal expression patterns of *Inhba* (B) and *Angpt2* (C) in the DRGs of young adult rats at 0, 1, 4, and 7 days post sciatic nerve injury in this study. *Angpt1*: Angiopoietin 1; *Angpt2*: angiopoietin 2; *Areg*: amphiregulin; *Bdnf*: brain derived neurotrophic factor; *Bmp6*: bone morphogenetic protein 6; *DRG*: dorsal root ganglia; *Fgf15*: fibroblast growth factor 15; *Fgf18*: fibroblast growth factor 18; *Fgf3*: fibroblast growth factor 3; *Fgf9*: fibroblast growth factor 9; *Gas6*: growth arrest specific 6; *Gdf3*: growth differentiation factor 3; *Gdf7*: growth differentiation factor 7; *Grn*: granulin precursor; *Grp*: gastrin releasing peptide; *Inhba*: inhibin subunit beta A; *Jag1*: jagged canonical notch ligand 1; *Jag2*: jagged canonical notch ligand 2; *Mstn*: myostatin; *Nell1*: neural EGFL like 1; *Nog*: noggin; *Nrg1*: neuregulin 1; *Ntf3*: neurotrophin 3; *Ogn*: osteoglycin; *Pgf*: placental growth factor; *Ptn*: pleiotrophin; *Sostdc1*: sclerostin domain containing 1; *TGF*: transforming growth factor; *Tgfb1*: transforming growth factor beta 1; *Vgf*: VGF nerve growth factor inducible.

In view of the critical roles of growth factors in axonal growth (Poitras and Zochodne, 2022; Moya-Alvarado et al., 2023), we sought to analyze growth factor genes among the DEGs in the injured DRGs of young adult and aged mice using Ingenuity Pathway Analysis software. Some of these DEGs exhibited similar expression trends in the injured DRGs of both young adult and aged mice. For instance, compared with the group of young adult mice, noggin (*Nog*), fibroblast growth factor 9 (*Fgf9*), and neuregulin 1 (*Nrg1*) remained downregulated, while Bdnf, VGF nerve growth factor inducible (*Vgf*), amphiregulin (*Areg*), and fibroblast growth factor 3 (*Fgf3*) remained upregulated in DRGs of young adult and aged mice at 1 day post-SNI. Other such genes, including the TGF-β-related genes *Inhba* and *Angpt2*, were differentially expressed in either young adult or aged mice (**Figure 1A**; Zhou et al., 2022). Sequencing data showed that, consistent with mouse data, at 1 day post SNI, the DRG expression levels of *Inhba* and *Angpt2* were elevated compared with those in uninjured 0-day control rats. At later time points post SNI, the expression levels of *Inhba* and *Angpt2* remained higher than those of the uninjured control (**Figure 1B** and **C**; Gong et al., 2016).

### ActA and Ang2 support neurite outgrowth and axonal elongation in dorsal root ganglia neurons

**Figure 2:**
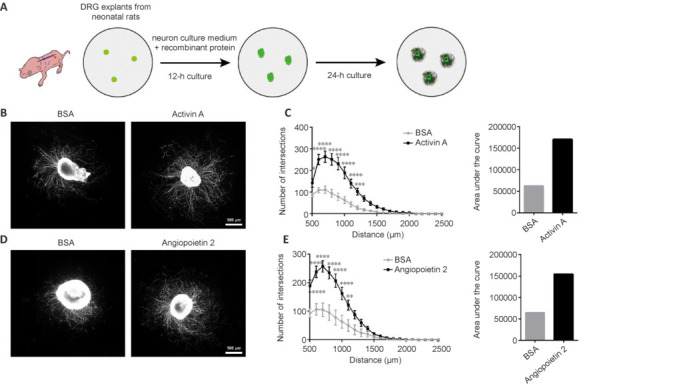
Neurite outgrowth in DRG explants following the administration of activin A or angiopoietin 2 protein. (A) Schematic illustration of the collection and treatment of neonatal rat DRG explants. Created with Adobe Photoshop CS5 and Adobe Illustrator CS6. (B, D) Representative Tuj1 immunostaining of DRG explants following treatment with recombinant activin A (B) or angiopoietin 2 (D), both of which promoted neurite growth, *vs*. BSA control. Scale bars: 500 μm. (C, E) Quantification of axonal intersections against the distance from the explant body and extrapolation of the area under the curve. Data analyzed using two-way analysis of variance followed by Sidak’s multiple comparisons test for the quantified number of axonal intersections. In C, 17 BSA-treated and 16 activin A-treated DRG explants from a total of six independent neonatal rats were examined; *adjusted *P* < 0.05, *P* = 0.0345; ***adjusted *P* < 0.001, *P* = 0.0004; ****adjusted *P* < 0.0001. In E, 18 BSA-treated and 21 angiopoietin 2-treated DRG explants from a total of six independent neonatal rats were examined; **P < 0.01, *P* = 0.0041; ****P < 0.0001. BSA: Bovine serum albumin; DRG: dorsal root ganglia.

DRG explants from neonatal rats were isolated, incubated with cell culture medium containing rActA or rAng2 proteins, fixed, and stained with the neuronal marker Tuj1 (**Figure 2A**). The administration of rActA protein led to obvious increases in both the number and length of neurites growing and extending from DRG somata compared with BSA-treated DRG explants (**Figure 2B**). Quantification analysis showed that, in rActA-treated DRG explants, the number of neurites, especially those crossing the ring at 500 μm from the DRG somata, were elevated by 1.64 fold. The numbers of neurites crossing the ring at longer distances, particularly within the 600–1200 μm range from the DRG somata, were significantly more elevated. Additionally, the extrapolated AUC, an index of axonal growth, increased by **~**2.74 fold compared with the BSA control group (**Figure 2C**). Similarly, the administration of rAng2 elicited an obvious increase in the numbers of intersections at multiple positions, particularly in the 500–1100 μm range from the DRG somata, as well as the calculated surface AUC (**Figure 2D** and **E**). These findings directly demonstrated the promotional effects of rActA and rAng2 on the growth of neurites from the explant body.

**Figure 3:**
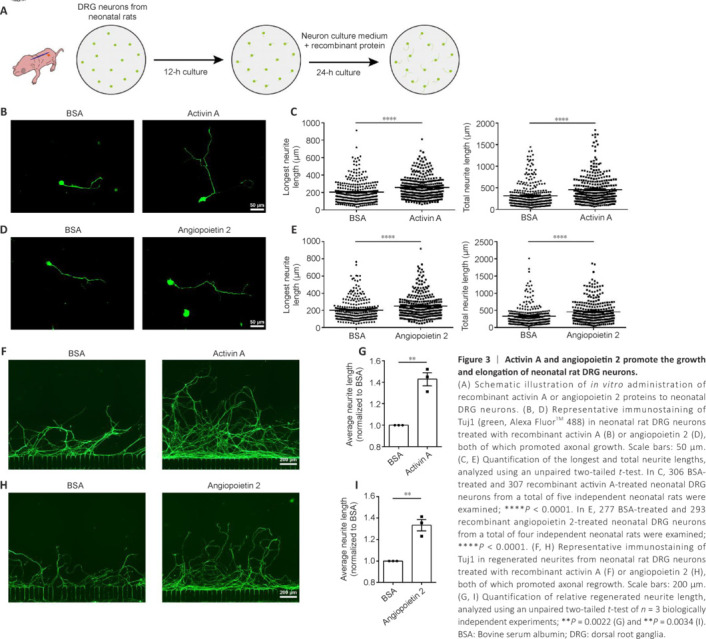
Activin A and angiopoietin 2 promote the growth and elongation of neonatal rat DRG neurons. (A) Schematic illustration of *in vitro* administration of recombinant activin A or angiopoietin 2 proteins to neonatal DRG neurons. (B, D) Representative immunostaining of Tuj1 (green, Alexa Fluor^TM^ 488) in neonatal rat DRG neurons treated with recombinant activin A (B) or angiopoietin 2 (D), both of which promoted axonal growth. Scale bars: 50 μm. (C, E) Quantification of the longest and total neurite lengths, analyzed using an unpaired two-tailed *t*-test. In C, 306 BSA-treated and 307 recombinant activin A-treated neonatal DRG neurons from a total of five independent neonatal rats were examined; ****P < 0.0001. In E, 277 BSA-treated and 293 recombinant angiopoietin 2-treated neonatal DRG neurons from a total of four independent neonatal rats were examined; ****P < 0.0001. (F, H) Representative immunostaining of Tuj1 in regenerated neurites from neonatal rat DRG neurons treated with recombinant activin A (F) or angiopoietin 2 (H), both of which promoted axonal regrowth. Scale bars: 200 μm. (G, I) Quantification of relative regenerated neurite length, analyzed using an unpaired two-tailed *t*-test of *n* = 3 biologically independent experiments; **P = 0.0022 (G) and **P = 0.0034 (I). BSA: Bovine serum albumin; DRG: dorsal root ganglia.

Next, we treated cultured DRG neurons of neonatal rats with rActA or rAng2 proteins (**Figure 3A**). The rActA significantly increased both the longest and total neurite lengths of cultured neonatal DRG neurons. Specifically, the longest neurites increased from 204.7 ± 7.399 μm in the BSA control group to 258.7 ± 7.464 μm in the rActA group, while the total neurite length increased from 319.4 ± 15.51 μm in the BSA control group to 459.1 ± 19.05 μm in the rActA group (**Figure 3B** and **C**). Exposure to rAng2 extended the longest neurites and total neurites from 203.2 ± 7.730 μm and 333.0 ± 17.43 μm, respectively, in the BSA control group to 250.0 ± 8.217 μm and 456.4 ± 18.92 μm in the rAng2 group (**Figure 3D** and **E**).

To explore the biological functions of these factors in axonal regeneration, we tested an *in vitro* model of axonal injury, in which axonal regeneration can be reliably quantified (**Figure 3A**). Neonatal DRG neurons displayed some regeneration capacity, with axons regrowing to **~**800 μm at 36 hours post axonal transection. The presence of rActA or rAng2 supported injured axons in extending much farther, enhancing the average neurite length by **~**40% beyond that of the BSA control group (**Figure 3F–I**).

**Figure 4:**
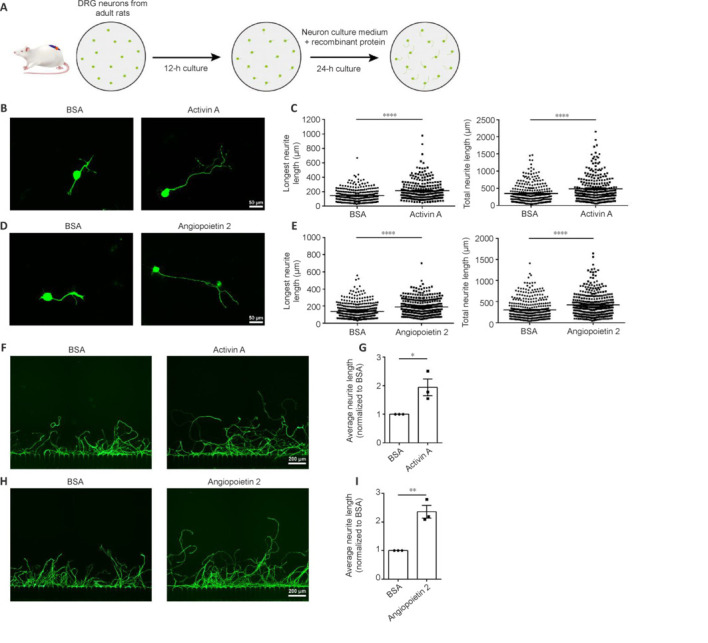
Activin A and angiopoietin 2 promote the growth and elongation of young adult rat DRG neurons. (A) Schematic illustration of *in vitro* administration of recombinant activin A or angiopoietin 2 proteins to young adult DRG neurons. (B, D) Representative immunostaining of Tuj1 (green, Alexa Fluor^TM^ 488) in young adult rat DRG neurons treated with recombinant activin A (B) or angiopoietin 2 (D), both of which promoted axonal growth. Scale bars: 50 μm. (C, E) Quantification of the longest and total neurite lengths, analyzed using an unpaired two-tailed *t*-test. In C, 279 BSA-treated and 267 recombinant activin A-treated adult DRG neurons from a total of five independent adult rats were examined; ****P < 0.0001. In E, 395 BSA-treated and 343 recombinant angiopoietin 2-treated adult DRG neurons from a total of five independent adult rats were examined; ****P < 0.0001. (F, H) Representative immunostaining of Tuj1 in regenerated neurites from adult rat DRG neurons treated with recombinant activin A (F) or angiopoietin 2 (H), both of which promoted axonal regrowth. Scale bars: 200 μm. (G, I) Quantification of relative regenerated neurite length, analyzed using an unpaired two-tailed *t*-test of *n* = 3 biologically independent experiments; *P = 0.0312 (G) and **P = 0.0035 (I). BSA: Bovine serum albumin; DRG: dorsal root ganglia.

The promotional effects of rActA or rAng2 treatment on neurite growth were also observed in cultured DRG neurons of young adult rats (**Figure 4A**). Exposure to either rActA or rAng2 increased the longest and total neurite lengths of young adult rat DRG neurons (**Figure 4B–E**). In the *in vitro* axonal injury system, injured young adult DRG neurons from the BSA control group exhibited diminished regeneration capacity, regrowing to approximately half of the neurite length of injured neonatal DRG neurons. By contrast, the administration of rActA or rAng2 strongly enhanced axonal regrowth ability, significantly increasing the average neurite length (**Figure 4F–I**). These findings indicated that ActA and Ang2 are capable of potentiating the axonal growth of adult neurons and may function as neuronal growth stimulators in adult mammals.

### ActA and Ang2 facilitate sensory axonal regeneration after sciatic nerve injury

**Figure 5:**
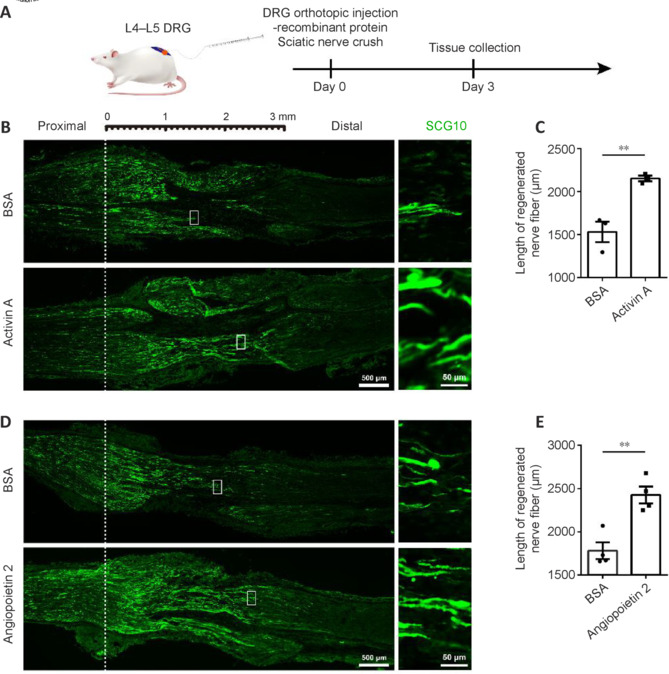
Activin A and angiopoietin 2 accelerate the regeneration of axons in young adult rats after SNI. (A) Schematic illustration of the *in vivo* DRG injection of BSA control or recombinant activin A or angiopoietin 2 into rats with SNI. Created with Adobe Photoshop CS5 and Adobe Illustrator CS6. (B, D) Representative immunostaining of SCG10 (green, Alexa Fluor^TM^ 488) in regenerated axons of young adult rats at 3 days post SNI with activin A (B) or angiopoietin 2 (D) treatment, both of which facilitated the regeneration of axons. Boxed areas are enlarged on the right. Scale bars: 500 μm (left) and 50 μm (right). (C, E) Quantification of the length of regenerated nerve fibers, analyzed using an unpaired two-tailed *t*-test. In C, data represent three biologically independent experiments; **P = 0.0073. In E, data represent four biologically independent experiments; **P = 0.0034. BSA: Bovine serum albumin; DRG: dorsal root ganglia; SCG10: Stathmin-2/STMN2; SNI: sciatic nerve injury.

Next, to evaluate the *in vivo* roles of ActA and Ang2, the recombinant proteins were directly applied to the DRGs of rats and the lengths of SCG10-positive regenerated sensory axons were compared at 3 days post sciatic nerve crush injury (**Figure 5A**). In young adult (8-week-old) rats, the L4–L5 injection of rActA induced a significant increase in the extension of injured nerve fibers, enhancing the length of SCG10-positive regenerated axons by 1.40 fold relative to the BSA control (**Figure 5B** and **C**). Likewise, rAng2 boosted the regeneration speed of injured axons, achieving a 36% increase in the length of the longest regenerated nerve fibers (**Figure 5D** and **E**).

**Figure 6:**
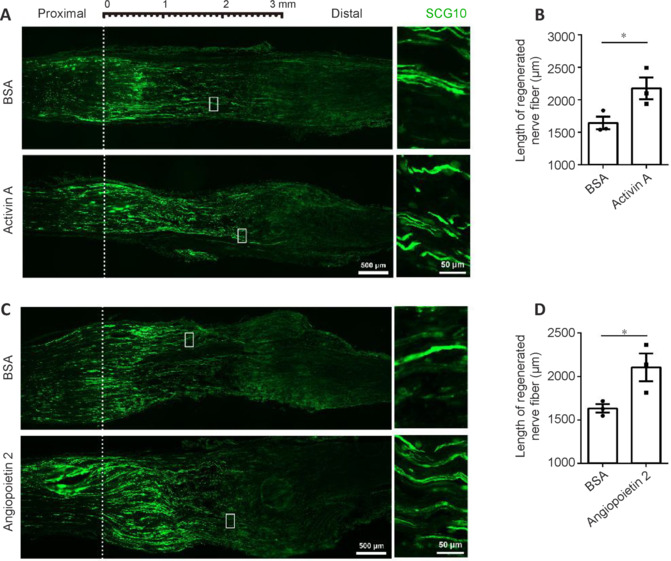
Activin A and angiopoietin 2 accelerate the regeneration of axons in middle-aged rats after SNI. (A, C) Representative immunostaining of SCG10 (green, Alexa Fluor^TM^ 488) in regenerated axons from middle-aged rats at 3 days post SNI and treatment with recombinant activin A (A) or angiopoietin 2 (C), both of which facilitated the regeneration of axons. Boxed areas are enlarged on the right. Scale bars: 500 μm (left) and 50 μm (right). (B, D) Quantification of the length of regenerated nerve fibers, analyzed using an unpaired two-tailed *t*-test of three biologically independent experiments; *P = 0.0499 (B) and *P = 0.0470 (D). BSA: Bovine serum albumin; DRG: dorsal root ganglia; SCG10: Stathmin-2/STMN2; SNI: sciatic nerve injury.

Comparable promotional effects of rActA and rAng2 on the regeneration of injured nerves were observed in middle-aged (10-month-old) rats. The rActA-treated middle-aged rats showed a higher average length of regenerated nerve fibers 2175 ± 165.4 μm compared with BSA-treated middle-aged rats (1644 ± 96.06 μm; **Figure 6A** and **B**). Injection of rAng2 to the DRGs of middle-aged rats also increased the length of regenerated nerve fibers from 1631 ± 49.15 μm in the BSA control group to (2105 ± 159.6 μm; **Figure 6C** and **D**). Taken together with the findings in isolated DRG explants and cultured DRG neurons, our results have highlighted the pro-regenerative roles of ActA and Ang2 in axonal regeneration, suggesting their therapeutic potential in the treatment of peripheral nerve injury.

### ActA promotes axonal regeneration by upregulating Gper1 and activating ERK signaling

**Figure 7:**
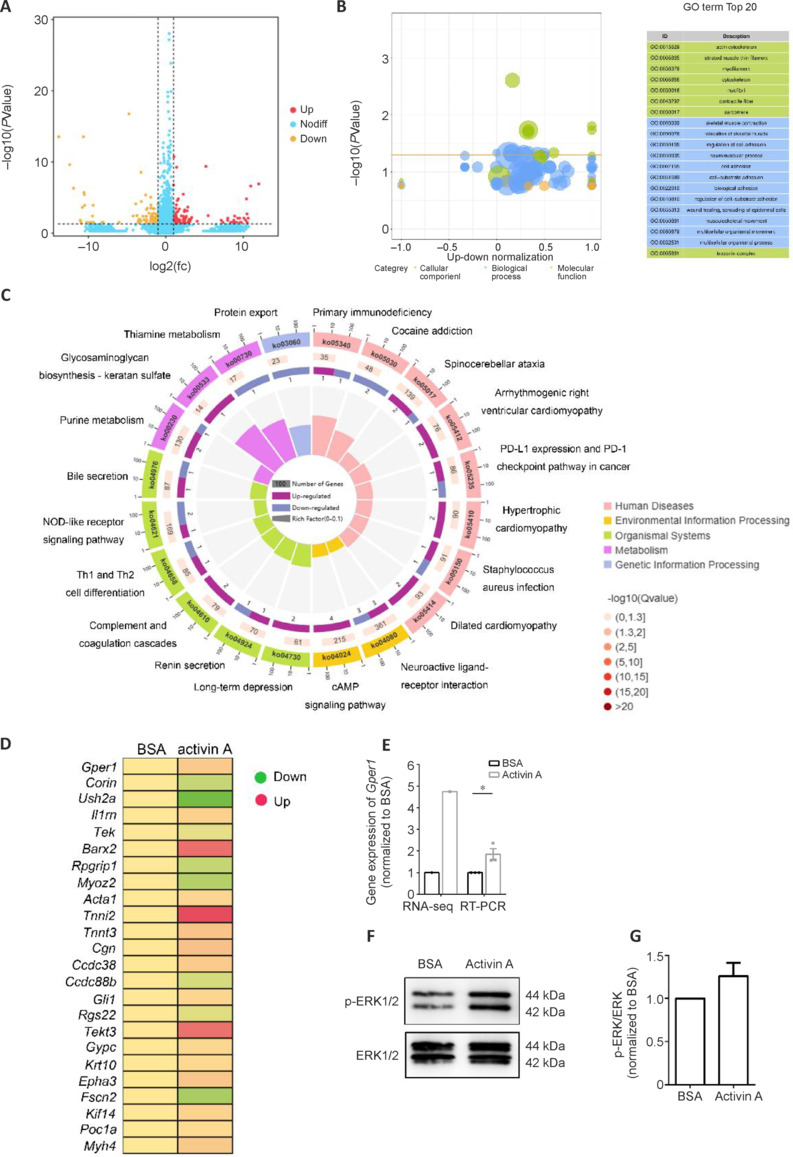
Activin A regulates axon skeleton-related genes in DRG neurons. (A) Scatter plots of DEGs in young adult DRG neurons treated with BSA control or recombinant activin A. (B, C) Top enriched GO terms (B) and KEGG pathways (C) of DEGs in recombinant activin A-treated DRG neurons. (D) Expression patterns of DEGs related to the cytoskeleton. (E) Gene expression level of Gper1 in DRG neurons treated with BSA or recombinant activin A, analyzed using an unpaired two-tailed *t*-test; *n* = 3 biologically independent experiments; *P < 0.05, *P* = 0.0316. (F) Protein expression level of phosphorylated (p)ERK1/2 and total ERK in DRG neurons treated with BSA or recombinant activin A. (G) Quantification of the ratio of pERK1/2 to total ERK (*n* = 3). BSA: Bovine serum albumin; DEGs: differentially expressed genes; DRG: dorsal root ganglia; ERK1/2: p44/42 mitogen-activated protein kinases (MAPK); GO: Gene Ontology; Gper1: G protein-coupled estrogen receptor 1; KEGG: Kyoto Encyclopedia of Genes and Genomes.

To decipher the molecular mechanisms underlying the promotional effects of TGF-β-related proteins ActA and Ang2 on axonal regeneration, we used RNA sequencing to examine the gene expression profiles of cultured DRG neurons from young adult rats treated with rActA or rAng2. We identified a total of 206 DEGs between BSA- and rActA-treated DRG neurons: 110 upregulated genes and 96 downregulated genes (**Figure 7A**). These DEGs were mainly involved in GO cellular component ontologies, particularly those related to the cytoskeleton (**Figure 7B** and **D**). Consistently, the cytoskeleton-related gene *Gper1* was found to be significantly upregulated following rActA exposure (**Figure 7E**). KEGG enrichment analysis showed that rActA also mediated the activation of signaling molecules and signal transduction, including the cAMP signaling pathway and neuroactive ligand–receptor interaction, in DRG neurons (**Figure 7C**).

GPER1, upon binding ligand binding, is capable of stimulating adenylate cyclase, increasing intracellular cAMP levels, and motivating cAMP-dependent ERK signaling (Khan et al., 2015). Hence, we examined the phosphorylation status of ERK1/2 in rActA-treated DRG neurons. This revealed that rActA exposure upregulated the amount of phosphorylated (p)ERK1/2, elevating the ratio of pERK1/2 to total ERK1/2 by **~**1.25 fold, indicating that ERK signaling is activated in DRG neurons treated with rActA (**Figure 7F**).

### Ang2 promotes axonal regeneration by upregulating transcription factor gene *E2f2*

**Figure 8:**
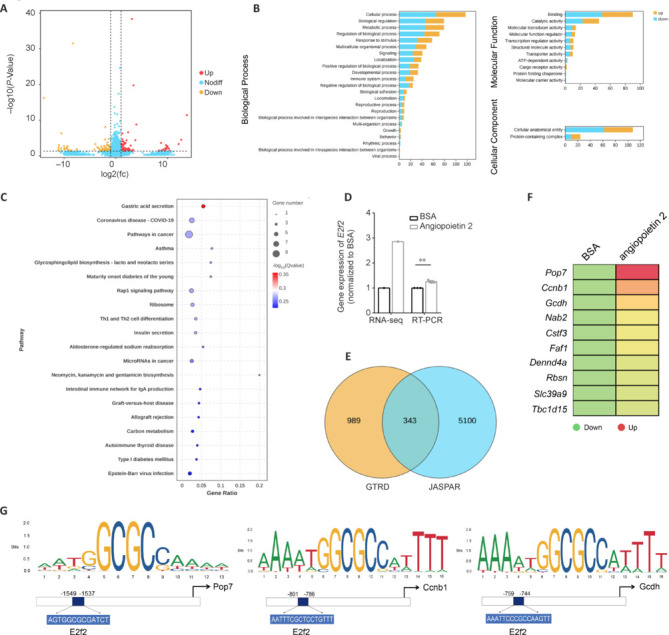
Angiopoietin 2 regulates cancer pathway-associated genes in DRG neurons. (A) Scatter plots of DEGs in young adult DRG neurons treated with BSA control or recombinant angiopoietin 2. (B, C) Enriched GO terms (B) and KEGG pathways (C) of DEGs in recombinant angiopoietin 2-treated DRG neurons. (D) Relative gene expression level of *E2f2* in DRG neurons treated with BSA or recombinant angiopoietin 2, analyzed using an unpaired two-tailed *t*-test; *n* = 3 biologically independent experiments; **P < 0.01, *P* = 0.0025. (E) Venn diagram of the number of predicted target genes of transcription factor E2F2 in two databases. (F) Candidate E2F2 target genes that were upregulated after recombinant angiopoietin 2 treatment. (G) Sequence logo plots and putative E2F2 binding sites in the promoter regions of potential target genes *Pop7*, *Ccnb1*, and *Gcdh*. BSA: Bovine serum albumin; *Ccnb1*: cyclin B1; DEGs: differentially expressed genes; DRG: dorsal root ganglia; *Gcdh*: glutaryl-CoA dehydrogenase; GO: Gene Ontology; KEGG: Kyoto Encyclopedia of Genes and Genomes; *Pop7*: POP7 homolog ribonuclease P/MRP subunit.

Compared with BSA control, we found that rAng2 injection led to significant upregulation of 96 genes and significant downregulation of 111 genes in DRG neurons from young adult rats (**Figure 8A**). GO functional classification showed that these DEGs were primarily associated with biological process categories, particularly cellular processes, biological regulation, and metabolic processes (**Figure 8B**). KEGG analysis showed the enrichment of many disease-related signaling pathways, particularly those involved in cancer (**Figure 8C**). A total of nine candidate genes annotation with pathways in cancer displayed diverse expression patterns following rAng2 exposure, including wingless-type MMTV integration site family member 3 (*Wnt3*), lysophosphatidic acid receptor 4 (*Lpar4*), interleukin 7 (*Il7*), interleukin 15 (*Il15*), delta (*Dll*), collagen type IV alpha (*Col4a*), RAS guanyl-releasing protein 3 (*Rasgrp3*), classical protein kinase C gamma type (*Prkcg*), and *E2f2*. RT-PCR analysis showed that gene expression of *E2f2*, which encodes a member of the E2F family of transcription factors, was indeed elevated in rAng2-treated neurons (**Figure 8D**).

GTRD and JASPAR-based screening for potential target genes of E2F2 predicted 343 intersecting genes (**Figure 8E**). Of these, a total of 10 candidate genes showed a similar expression trend to *E2f2*. All 10 were upregulated following rAng2 treatment, with *Pop7*, *Ccnb1*, and *Gcdh* showing the most significant upregulation (**Figure 8F**). Bioinformatic analysis showed putative E2F2 binding sites in the promoter regions of Pop7 (–1549 to –1537 bp), Ccnb1 (–801 to –786 bp), and Gcdh (–759 to –744 bp), suggesting that E2F2 may regulate the expression of these downstream targets (**Figure 8G**).

## Discussion

Reversing the aging process and recapitulating neuronal developmental processes may enhance the growth competency of adult neurons and accelerate the repair of injured nerves (Mahar and Cavalli, 2018). TGF-β is a key regulator of multiple physiological and pathological processes, including peripheral nerve regeneration (Li et al., 2017; Ding et al., 2024). Elevated TGF-β expression in injured peripheral nerves shifts Schwann cells to an invasive mesenchymal-like phenotype and facilitates the generation of a microenvironment permissive for axonal elongation (Clements et al., 2017; Jessen and Arthur-Farraj, 2019). Besides mediating Schwann cell reprogramming, TGF-β can promote axonal regeneration by directly stimulating the intrinsic regenerative ability of DRG neurons (Chandran et al., 2016). The addition of TGF-β to DRG explants remarkably enhances the outgrowth of neurites from neuronal somas, achieving an even better effect than nerve growth factor (Au et al., 2007).

Herein, we observed that, similar to treatment with recombinant TGF-β protein, the addition of rActA or rAng2 to DRG explants led to noticeable increases in the length and number of neurites growing from neuronal somas. In addition, we showed that the application of rActA or rAng2 supported the growth of neuronal neurites in neonatal rat DRG neurons, which exhibit strong growth ability, as well as young adult rat DRG neurons, which have relatively lower growth ability. These observations expand our understanding of the biological roles of TGF-β family members and demonstrate that other members of the TGF-β superfamily, as well as TGF-β-related growth factors, may also encourage axonal growth.

Using a compartmentalized microfluidic device-based *in vitro* axonal injury model and an *in vivo* peripheral nerve injury model, we directly demonstrated the promotional effects of ActA and Ang2 on axonal elongation and nerve regeneration. In the animal studies, rActA or rAng2 was directly injected into the DRGs immediately after rat SNI, with the delivery of either protein sufficient to enhance axonal growth in both 8-week-old (young adult) and 10-month-old (middle-aged) rats. Although the axonal regeneration capacity is generally considered to decrease with aging, there were no significant differences in the length of regenerated nerve fibers between young adult and middle-aged rats, regardless of the addition of rActA or rAng2. This finding indicates that the decline of axonal growth competency in middle age is slight, with middle-aged animals possessing regenerative capacity comparable to young adult animals following peripheral nerve injury.

Notably, although rActA and rAng2 exhibited comparable promotional effects on axonal regeneration, the two proteins induced distinct changes in genes associated with different GO and KEGG functional classifications. After treatment with rActA, actin cytoskeleton- and cytoskeleton-related GO terms were significantly enriched. The cytoskeleton is important for regeneration (Li et al., 2023; Alsina et al., 2024). RNA sequencing and RT-PCR results demonstrated that rActA treatment significantly upregulated the expression of cytoskeleton-related *Gper1* in young adult DRG neurons. In our study, activin A increases the gene expression of cytoskeleton-related gene Gper1 and the protein expression of pERK1/2. GPER1 is an estrogen receptor whose stimulation elicits the activation of cAMP signaling and its downstream ERK cascades (Stork and Schmitt, 2002; Khan et al., 2015). ERK signaling regulates genes that controls cytoskeleton dynamics and mediates axonal growth during development and regeneration (Hausott et al., 2022). The expression of GPER1 in the nociceptive pathway, including DRG neurons, has been demonstrated (Sarajari and Oblinger, 2010). Furthermore, the expression of GPER1 in DRG neurons is elevated following sciatic nerve crush injury or spared nerve injury (Xu et al., 2022; Zhao et al., 2023). The activation of GPER1 in DRG neurons reportedly leads to increased expression of the anti-apoptotic Bcl-x and enhances neuronal survival (Patrone et al., 1999). Our findings imply that GPER1, acting downstream of ActA, may also contribute to axonal regeneration, and that ActA may enhance the intrinsic growth ability of DRG neurons by upregulating GPER1 expression and activating ERK signaling.

Ang2 mediates the genetic changes of many cancer-related molecules. Given that transcription factors are essential regulators of axonal regeneration (Zhang et al., 2023), we screened for DEGs encoding transcription factors among the enriched cancer-related pathways, identifying upregulation of transcription factor gene *E2f2* in neurons following rAng2 exposure. E2F2 has been demonstrated to be a positive regulator of axonal growth, particularly when combined with a CCCTC-binding factor (Avraham et al., 2022). Here, we discovered that Ang2 may contribute to axonal regeneration by increasing the abundance of E2F2. Furthermore, by integrating the predicted downstream *targets* of E2F2 with rAng2-mediated DEGs, we identified *Pop7*, *Ccnb1*, and Gcdh as potential targets of axonal growth and nerve regeneration.

Our comparison of the genetic changes underlying the differential regenerative responses to nerve injury in young adult and aged animals demonstrated that the TGF-β-related growth factor genes *Inhba* and *Angpt2* were elevated in the injured DRGs of young adult animals but not differentially expressed in aged animals. We also characterized the biological functions of ActA and Ang2 proteins, concluding that each plays a promotional role in axonal regeneration and nerve repair. The failure to elevate ActA and Ang2 levels in the injured DRGs of aged animals may contribute to the age-related diminishment of intrinsic neuronal regeneration properties.

Here, we explored the biological effects of rActA and rAng2 on axonal growth and elongation using dissociated DRG explants, cultured DRG neurons, and adult animals, identifying these two TGF-β-related proteins as positive regulators of axonal regeneration. Mechanistically, we found that ActA induces transcriptomic regulation of axon cytoskeleton-related genes, such as *Gper1*, and activates ERK1/2 phosphorylation, while Ang2 engages distinct biological processes, particularly pathways in cancer, and mediates the molecular changes of transcription factor E2F2.

By revealing the functional roles of TGF-β-related proteins ActA and Ang2 in isolated DRG explants, cultured DRG neurons, and peripheral nerve-injured rats, our study has clarified the importance of ActA and Ang2 in enhancing the regeneration capacity of neurons, offering a novel approach to promoting axonal regeneration. Future studies could further explore post-SNI axonal growth through additional animal experiments, including analysis of multiple time points (e.g., long-term recovery) along with cross-sectional immunostaining of the sciatic nerve. Additionally, axonal regeneration could be assessed using electron microscopy and behavioral tests (e.g., thermal and mechanical pain). One limitation of this study is that experimental constraints prohibited us from treating aged (20-month-old) rats with rActA or rAng2 proteins. Aged rats have poor tolerance to surgery, relatively long postoperative recovery time, and significantly increased mortality rate. At the same time, it is necessary to ensure that experimental procedures comply with animal welfare standards and reduce pain, thus requiring more frequent pain assessments and interventions. We will optimize the experimental conditions and use aged rats as the animal experimental model subjects in the future. The possibility that these TGF-β-related growth factors could enhance nerve regeneration in aged animals warrants further investigation. In future research, we plan to explore the promotional effects of these growth factors on axonal growth in both adult and aged rats, which may provide even more valuable insights.

## Data Availability

*All relevant data are within the paper*.
